# Oncogene-mediated regulation of p53 ISGylation and functions

**DOI:** 10.18632/oncotarget.2199

**Published:** 2014-07-11

**Authors:** Yi-Fu Huang, Dmitry V. Bulavin

**Affiliations:** ^1^ Institute of Molecular and Cell Biology, Proteos, Singapore; ^2^ Institute for Research on Cancer and Ageing of Nice (IRCAN), INSERM, U1081-UMR CNRS 7284, University of Nice - Sophia Antipolis, Centre Antoine Lacassagne, Nice, France

**Keywords:** p53, ISG15, cancer, protein degradation

## Abstract

Oncogene-mediated cellular transformation is a multistep process involving activation of growth-promoting pathways as well as inactivation of tumor suppressors. We recently found that ISGylation of the p53 tumor suppressor is an important novel mechanism to control its stability. Here we identified that Isg15-dependent regulation of p53 can be enhanced by different oncogenes. We further show that the Src-mediated phosphorylation of p53 on Tyr126 and Tyr220 has a positive effect on p53 ISGylation by enhancing Herc5 binding. In turn, deletion of Isg15 results in accumulation and activation of native p53 in transformed cells thus increasing its anti-cancer activity and suppressing tumorigenesis in mice. We propose that Isg15-dependent degradation of p53 is an alternative pathway for oncogenes to regulate p53 activity, and thus is an attractive pathway for development of new anti-cancer drugs.

## INTRODUCTION

The p53 transcription factor is a key tumor suppressor. The p53 activity is required to activate cell cycle arrest, apoptosis, and senescence in order to prevent tumorigenesis, while its inactivation is important for oncogene-mediated transformation. Controlling the stability of the p53 tumor suppressor through polyubiquitination and subsequent degradation by the 26S proteasome is central to the regulation of p53 functions. We recently found that in addition to degradation of p53 through the ubiquitin-26S proteasomes-dependent system, it can be modified by Isg15 creating a degradation signal for the 20S proteasomes [1]. This degradation pathway in normal cells, when the Isg15 system is relatively inefficient, appeared to be targeting primarily misfolded, dominant-negative p53. As a consequence, deletion of Isg15 resulted in accumulation of misfolded p53 reducing the overall p53 activity in primary cells including fibroblasts, neural stem/progenitor cells and thymocytes [1].

Isg15, the product of IFN-stimulated gene 15, was the first reported ubiquitin-like protein[2]. Isg15 is robustly induced by different stimuli, including type 1 IFNs, lipopolysaccharide, viruses, and oncogenes [3-6]. Similar to ubiquitin conjugation, ISGylation of target proteins occurs in a three-step cascade mechanism that involves Isg15-activating E1 enzyme (UBE1L), Isg15-conjugating E2 enzyme (UBCH8), and Isg15 E3 ligase with HERC5 being the main E3 ligase for Isg15 [6-10].

It is believed that the Isg15 system is relatively inefficient in normal cells due to its low expression levels. In contrast, Isg15 was found to be overexpressed in numerous primary human cancers, including endometrial tumors[11], pancreatic adenocarcinomas[12], tumors of the breast [13, 14], bladder[15], and prostate[16, 17]. In addition, its expression is known to be required for oncogene-mediated transformation. The exact role of Isg15 in oncogene-mediated transformation or in cancer however is still unclear.

In this study, we found that different oncogenes can efficiently induce p53 ISGylation. We further found that p53 Tyr 126 and Tyr220 were critically involved in Src-dependent p53 ISGylation through regulation of p53 binding with its E3 ligase, Herc5. Deletion of Isg15 in cancer cells increased both misfolded and native p53, which was in contrast to accumulation of only misfolded form in normal cells [1]. This in turn resulted in upregulation of p53 activity in transformed cells and suppression of tumorigenesis in mice. We propose that p53 ISGylation plays an important role in inactivation of p53 during oncogene-mediated transformation, and thus may be an attractive pathway for cancer therapy.

## RESULTS

### Oncogenes promote p53 ISGylation

**Figure 1:**
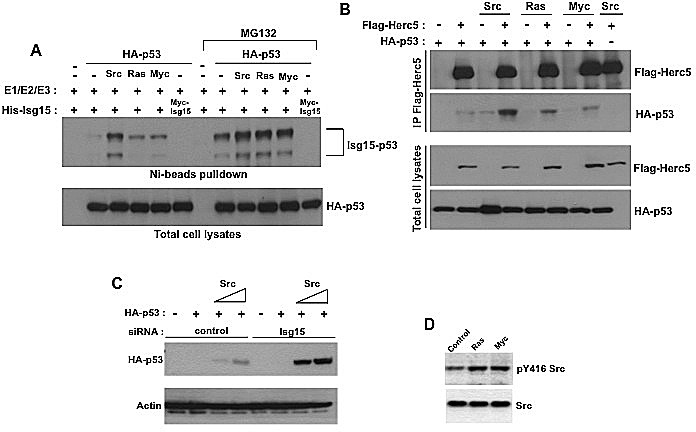
Different oncogenes enhance p53 ISGylation (A) HEK293T cells were transfected with p53, Isg15-modifying enzymes and difference oncogenes (Src, Ras, Myc). In some cases, proteosome inhibitor MG132 (25 μM) was added. After Ni-beads pulldown, Isg15-modified p53 was analyzed by Western blotting with 1801 antibody. (B) Different oncogenes increase the interaction between p53 and Herc5. HEK293T cells were transfected with HA-p53, Flag-Herc5 and difference oncogenes (Src, Ras, Myc). Flag-Herc5 was immunoprecipitated using anti-Flag M2-conjugated beads, and p53 in the complexes was detected by Western blotting. (C) Isg15 siRNA knockdown enhances Src-mediated p53 stabilization. HEK293T cells were transfected with HA-p53, Src, and control or Isg15 siRNAs. Cell lysates were analysed by Western blotting using indicated antibodies. (D) Overexpression of Ras or Myc increases Src phosphorylation as a read-out of its activity. HEK293T cells were transfected with Ras or Myc. The Src phosphorylation was analyzed by Western blotting using phospho-Src Tyr416 antibody.

In contrast to normal cells, Isg15 is highly expressed in numerous primary human cancers, and its expression is known to be required for oncogene-mediated transformation [11, 14, 16]. The exact role of Isg15 in oncogene-mediated transformation however is still unclear. Therefore, we explored the possibility that Isg15 in cancer cells acts through regulation of p53 as we previously found that Isg15 can efficiently target p53 for degradation [1]. We first tested whether different oncogenes can promote p53 ISGylation. Src, Ras, and Myc oncogenes were transfected, as these oncogenes commonly amplified and overexpressed in primary human cancers[18] and p53 ISGylation was analyzed. We found that overexpression of all three oncogenes increased their expression (Figure S1) and p53 ISGylation, with Src being the most efficient (Figure 1A). This could be because the oncogenes enhanced the interaction between p53 and the E3 ligase HERC5 (Figure 1B). Furthermore, knockdown of Isg15 significantly enhanced Src-induced accumulation of p53 (Figure 1C), suggesting that Isg15 is critically involved in Src-dependent regulation of p53. As overexpression and activation of Ras and myc in primary human cancers commonly results in activation of Src [18] and we further confirmed that (Figure 1D), for further analysis we concentrated on the role of Src in the regulation of p53 ISGylation.

### Isg15 depletion increases Src activity-induced accumulation of endogenous p53

**Figure 2:**
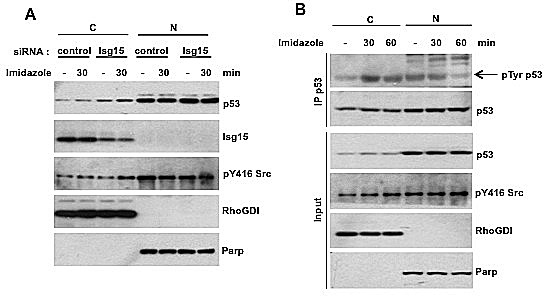
(A) Isg15 knockdown by siRNA enhances Src activity-mediated p53 stabilization. The Src mutant (8A7F, R388A/Y527F) was stably transfected in HCT116 cells with Flag-tagged endogenous p53. The cells were transfected with control or Isg15 siRNA. 48h after transfection, the cells were treated with imidazole (5 mM) to activate Src activity. Cytosolic (C) and Nuclear (N) fractions were then separated, and the level of p53 was analyzed by Western blotting. Parp and RhoGDI were used as nuclear and cytosolic markers, respectively. (B) The increase of Src activity by chemical rescue promotes p53 tyrosine phosphorylation. The Src mutant (8A7F, R388A/Y527F) was stably transfected in HCT116 cells with Flag-tagged endogenous p53. After treatment of MG132 (25 μM) for 1.5h, the cells were treated with imidazole (5 mM) to activate Src activity, and collected at the indicated times. Cytosolic (C) and Nuclear (N) fractions were then separated. p53 was immunoprecipitated using anti-flag M2 beads and analyzed with phospho-Tyrosine antibody. Parp and RhoGDI were used as nuclear and cytosolic markers, respectively.

The phosphorylation of proteins including p53 is known to contribute to their degradation by modulating binding with E3 ligases [19]. Because Src is a protein tyrosine kinase, we speculated that the effect of Src on p53 binding with Herc5 and ISGylation could be through phosphorylation. p53 is degraded both in the nucleous and cytoplasm [20] and thus can be directly targeted by Src. To verify this, we used an inducible system to activate Src [21] and analyzed the level of p53 phosphorylation on tyrosine. We found that depletion of Isg15 resulted in accumulation of p53 in cytoplasm (Figure 2A) as well as tyrosine phosphorylation was specifically increase on cytoplasmic p53 only after activating Src (Figure 2B). These results argue that Src can efficiently phosphorylate endogenous p53 in cytoplasm.

### Src-mediated phosphorylation enhances p53 ISGylation

**Figure 3:**
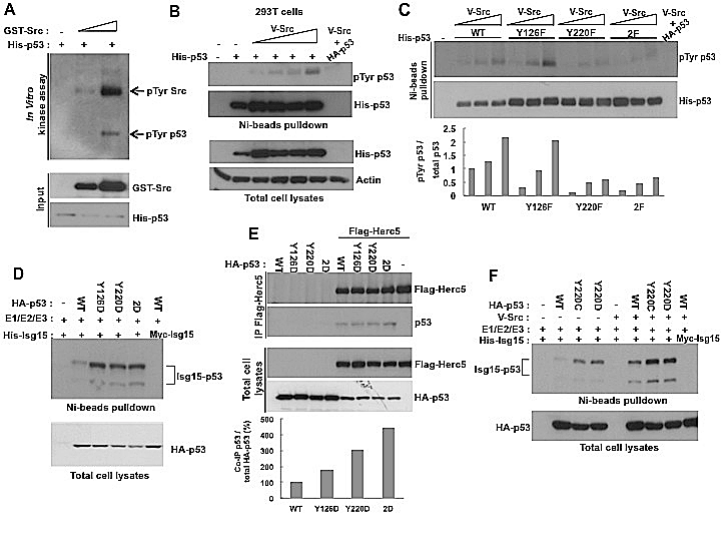
The phosphorylation of p53 on Tyr126 and Tyr220 promotes ISGylation (A) Src phosphorylates p53 *in vitro*. *In vitro* kinase assay was performed by incubating purified His-tagged p53 with Src. The products were analysed by Western blotting using anti-phospho-Tyrosine antibody. (B) Src increases Tysosine phosphorylation of p53 in HEK293T cells. His-p53 was co-transfected Src and analyzed by Western blotting with 1801 antibody after Ni-beads pulldown. (C) Src phosphorylates p53 at Tyr126 and Tyr220. HEK293T cells were transfected with Src and WT, Y126F, Y220F, or 2F (Y126F+Y220F) His-53. p53 was precipitated with Ni-beads and analyzed with phospho-Tyrosine antibody. The results were quantified by densitometry and analyzed by GelPro software (lower panel). (D) Phospho-mimicking mutations ofTyr126 and Tyr220 increases p53 ISGyaltion. HEK293T cells transfected with WT, Y126D, Y220D, or 2D (Y126D+Y220D) mutants of p53 were analyzed for p53 ISGylation after Ni-beads pull-down. (E) Phospho-mimicking mutations of Tyr126 and Tyr220 increase p53 interaction with Herc5. HEK293T cells were transfected with WT, Y126D, Y220D, or 2D (Y126D+Y220D) p53 mutants and Flag-Herc5. Flag-Herc5 was immunoprecipitated and p53 was analyzed by Western blotting and results were quantified by densitometry (lower panel). (F) Y220C mutation increases p53 ISGylation. HEK293T cells transfected with WT, Y220C, or Y220D p53 mutants together with Isg15-modifying enzymes were analyzed for p53 ISGylation after Ni-beads pull-down.

Next, we confirmed that p53 is directly phosphorylated by Src both *in vitro* and *in vivo* (Figure 3A&B). We identified two Src-conserved phosphorylation sites on p53, Tyr126 and Tyr220, and we found that mutation of Tyr220 to Phe (Y220F) or both sites (Y126F+Y220F) largely decreased the phosphorylation of p53 by Src (Figure 3C). To determine the effect of phosphorylation on p53 ISGylation, we generated phospho-mimicking mutants by substituting Tyr for Asp. We found that mutation of either site to Asp resulted in a significant increase in p53 ISGylation and promoted the interaction between p53 and Herc5 (Figure 3D&E). Another common cancer mutation, p53 Y220C, which results in destabilization of p53, also had an enhanced ability to be ISGylated (Figure 3F). Thus, our data argue that phosphorylation of p53 at Tyr126/220 or mutation of Tyr220 results in enhanced p53 ISGylation and degradation in cancer cells.

### Isg15 depletion increases both unfolded and folded p53 in transformation cells

**Figure 4:**
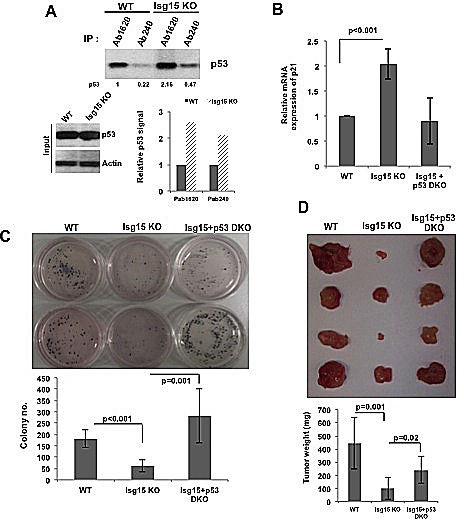
Isg15 regulates oncogenes-mediated transformation (A) Isg15 knockout increases unfolding and folding form of p53 in the transformed cells. Lysates from V-Src transformed mouse embryo fibroblasts (MEFs) (WT or Isg15 knockout) were immunoprecipitated with p53 antibodies Ab1620 or Ab240. The immunoprecipitated p53 was analysed by Western blotting. (B) Knockout of Isg15 increases the expression of *p21* gene in transformed cells. RT-PCR was performed to analyse the p21 expression of V-Src transformed WT or Isg15 knockout MEFs cells. (C&D) Isg15 knockout enhances p53-mediated inhibition of transformation. (C) V-Src transformed WT, Isg15 knockout, or Isg15/p53 double knockout MEFs were grown in soft agar. Colonies were stained with MTT and counted 3 weeks later. (D) Transformed MEFs were injected into NSG nude mice. Tumors were collected and analyzed 21d after injection.

Our previous data shows that deletion of Isg15 results in accumulation of misfolded form of p53 in primary cells. To investigate this in transformed cells, we next obtained mouse embryo fibroblasts (MEFs) from wild-type and Isg15-deficient mice and transformed them with Src oncogene. Next, we immunoprecipitated p53 with conformation-specific antibodies. The conformation of p53 can be assessed using Ab1620 antibody for wild-type p53 [22] and Ab240 for p53 in the unfolded or denatured conformation[23]. We found that in contrast to primary cells [1], deletion of Isg15 in transformed cells resulted in accumulation of both misfolded and native forms of p53 (Figure 4A). Analysis of p53 transcriptional activity showed a significant upregulation of p53 downstream target, p21/Waf1 mRNA, in Isg15-deficient Src-transformed cells in a p53-dependent manner (Figure 4B). Next, we analysed the colony-forming activity of Src-transformed MEFs and found that a deficiency of Isg15 significantly reduced the ability to form colonies in soft agar (Figure 4C). Importantly, this tumor-suppressor effect was p53 dependent as it was fully reversed by simultaneous deletion of p53 (Figure 4C). We further found a p53-dependent suppression of tumor growth after injecting Src-transformed Isg15-deficient cells into the NSG nude mice (Figure 4D). These data argue that in contrast to normal cells [1], deletion of Isg15 in transformed cells results in upregulation of p53 activity and functions.

### Isg15 deficiency suppresses K-ras-driven lung tumorigenesis

To understand the potential role of Isg15 in the regulation of tumorigenesis in vivo, we carried out the bioinformatics analysis of different types of human cancers in order to identify subsets with high level of active Src. Analysis of tumors from TCGA datasets [18] showed a strong accumulation of phospho-Src in lung squamous cell carcinoma and lung adenocarcinoma. As both types of cancer contain frequently mutated Ras allele and in turn Ras efficiently induces p53 ISGylation (Figure 1A&B), next we turned to the analysis of lung cancer in K-ras mice [24].

**Figure 5:**
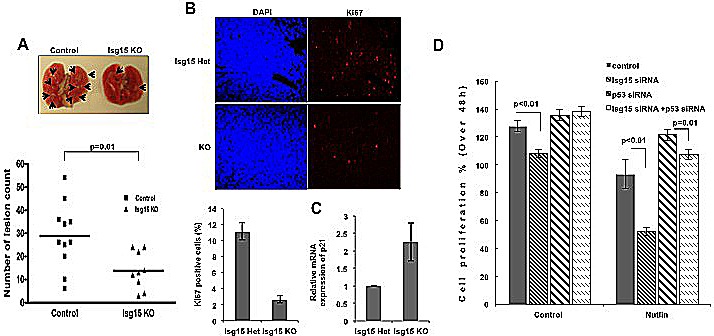
Knockout of Isg15 suppresses cancer formation (A) Isg15 knockout impairs K-ras-induced lung tumors. 10-week-old Kras mice (control mice, n=10 (including 5 WT and 5 Isg15 Het mice); Isg15 knockout mice, n=8) were sacrificed, and lung lesions were counted (shown with arrows). (B) Isg15 knockout decrease the proliferation of K-ras-induced lung cancer. The lung collected from Isg15 Het or Isg15 knockout K-ras mice was sectioned and stained with Ki67 antibody. The positive cells of Ki67 in tumor region were showed and quantified. (C) Knockout of Isg15 increases the expression of p21 gene in K-ras-induced lung cancer. The K-ras-induced tumors were dissected from lungs containing cancer lesions. RT-PCR was performed to analyse the p21 mRNA expression. (D) Knockdown of Isg15 enhances the Nutlin-mediated inhibition of cell proliferation. MTT assay were performed with MCF7 cells transfected with either control, Isg15 siRNA, p53 siRNA or a combination of siRNAs in the absence or presence of Nutlin (3 μM). The difference of proliferating activity between 24h to 72h after seeding was shown in graphs.

To assess the effects of Isg15 in vivo, we crossed Isg15^−/−^ and K-ras mice to evaluate the onset of lung tumorigenesis. Subsequently, the Isg15^+/−^ K-ras mice were intercrossed; K-ras and Isg15^−/−^ K-ras littermates were used for further evaluation. Our analysis of tumor formation in 10 weeks old mice revealed that the number of lesions of different sizes was significantly reduced in an Isg15-deficient background (Figure 5A). We further found that a deficiency of Isg15 significantly reduced the proliferation of cancer cells in K-ras lesions (Figure 5B) as well as upregulated p53 responsive gene, p21 (Figure 5C). These data argue that Isg15 controls K-ras-driven lung tumorigenesis in mice.

### Isg15 depletion enhances the Nutlin-3-mediated p53 activation in cancer cells

Next we turned to the functional analysis of p53 ISGylation in human cancer cells. First, we analyzed short-term effects of Isg15 knockdown on DNA replication based on scoring S-phase cells using EdU incorporation protocol. We found a significant reduction in EdU incorporation in HCT116 cells with siRNA-depleted Isg15 (Figure S2A). In turn, removal of p53 by siRNA reversed the effect of inhibition, suggesting that the effect of Isg15 knockdown on DNA replication was p53-dependent.

Ubiquitination and ISGylation of p53 have been reported to compete with each other for the lysine residue, and both depletion result in p53 activation. So, to understand whether the effect of Isg15 knockdown could be enhanced further by blocking p53 ubiquitination, we knocked down Isg15 levels with siRNA or blocked Mdm2-dependent degradation with nutlin-3 or used both treatments. We found that single treatments were efficient in reducing cell proliferation, however the effect was significantly potentiated when both ISGylation and polyubiquitination were inhibited (Figure 5D, S2B, S2C). In turn, removal of p53 by siRNA reversed the effect of inhibition, suggesting that the effect of Isg15 knockdown or Mdm2 inhibition is p53-dependent.

### Isg15 depletion increases DNA damage-induced p53 activity

**Figure 6:**
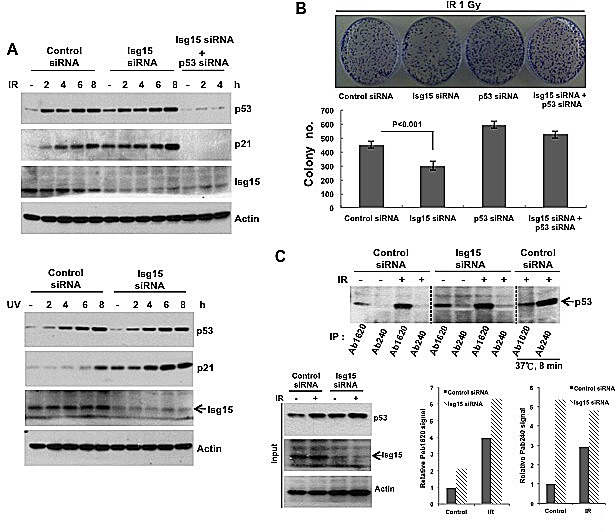
Isg15 regulates p53-dependent DNA damage response (A) Knockdown of Isg15 increases DNA damage-induced p53 response. HCT116 cells were transfected with control or Isg15 siRNAs, or together with p53 siRNA. Cells were irradiated with 8 Gy of IR (upper panel) or treated with UV (30 J/m^2^) (lower panel) and collected at the time points indicated. Cell lysates were analysed by Western blotting using indicated antibodies. (B) Knockdown of Isg15 increases DNA damage-induced inhibition of survival. HCT116 cells that transfected as described in (A) were processed in colony formation assay after treatment with 1Gy of IR. 3000 cells were plated for each condition. (C) Isg15 knockout increases unfolding and folding form of p53 in the Isg15 knockdown cells. HCT116 cells were transfected with control or Isg15 siRNAs, then were irradiated with 8 Gy of IR for 6h. The cell lysates were collected and immunoprecipitated with p53 antibodies Ab1620 or Ab240. For testing the specificity of the conformation-specific antibody, the lysates were incubated at 37°C for 8 min to denature folded p53.

To understand the role of Isg15-dependent modifications in regulation of p53 functions after DNA damage, next we treated cells with control and Isg15 siRNA and subsequently treated them with different forms of stress. We found that transcriptional activation of p53 based on analysis of p53 downstream target, p21/Waf1, was significantly upregulated in Isg15 siRNA-treated samples after treatment with IR (Figure 6A, top panel), UV (Figure 6A, bottom panel) and adriamycin (Figure S3A). The p53 upregulation after IR further resulted in reduced colonies-forming activity of cells with depleted Isg15, the effect that was dependent on the presence of p53 (Figure 6B). We further observed a similar upregulation activation of p53 based on the level of p21 mRNA in cells treated with Herc5 siRNA (Figure S3B). To understand whether this upregulation is resulted from accumulation of native p53 in Isg15 knockdown cells, p53 was immunoprecipitated with conformation-specific antibodies. We found Isg15 depletion increases both unfolded and native p53 in cancer cells after DNA damage (Figure 6C). Together, our data argue that Isg15-dependent modifications play a role in regulation of p53 functions under normal growth conditions and after DNA damage in cancer cells.

## DISCUSSION

Oncogenes-mediated cellular transformation is a multistep process involving activation of growth-promoting pathways and inactivation of tumor suppressors. Recent study showed that oncogenic tyrosine kinases Src and p210 ^bcr-abl^ can enhance the ubiquitination and degradation of Dok-1, the negative regulator of transformation, by phosphorylating Dok-1, and thus promoting cell transformation [25]. In this study, we found ISGylation-mediated degradation of p53 is another pathway for oncogenes to inhibit negative regulators in this transformation process.

**Figure 7:**
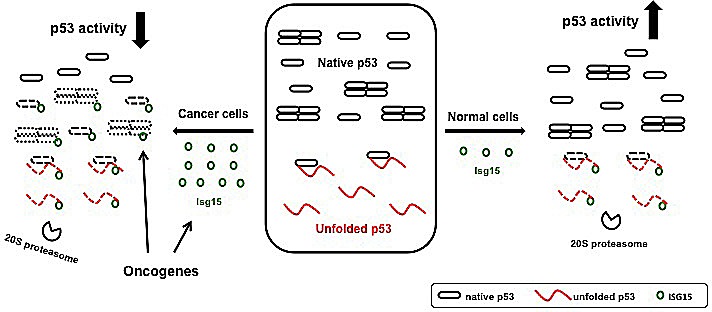
A model for Isg15-dependent degradation of p53 In normal cells, ISGylation primarily targets misfolded dominant-negative form of p53 thus promoting a total p53 activity (1). In cancer cells, the level of Isg15 is increased and the presence of different oncogenes promotes the interaction between p53 and Herc5 to increase the p53 ISGlyation. As a result, native p53 is targeted by Isg15-dependent degradation reducing the overall p53 activity.

In contrast to normal cells, the ISGylation system is activated in different types of cancer. Several mechanisms could contribute to this activation including upregulation of Isg15 levels. Isg15 was found to be increased in numerous primary human cancers, including endometrial tumors [11]; pancreatic adenocarcinomas[12]; tumors of the breast with Isg15 expression independent of HER2, progesterone, and estrogen receptor status [13, 14]; cancer of the bladder [15] and prostate [16]. In some cases, this upregulation was associated with enhanced metastasis and poor patient prognosis [13]. Importantly, in cancer cells p53 ISGylation can be further activated through modulation of p53 binding with E3 ligase Herc5 as we describe here for several oncogenes including Src. This results in Isg15-dependent degradation of p53 in a native wild-type confirmation in addition to misfolded p53 (Figure 4A&6C). Depletion of Isg15 in cancer cells, in turn, results in upregulation of p53 activity and functions (Figure 7).

To further support the role of Isg15 as a positive regulator of tumorigenesis, we found that knockout of Isg15 significantly attenuated cancer cell proliferation and tumorigenesis in a p53-dependent manner (Figure 4&5). We found that Isg15 depletion enhances the Nutlin-3-mediated p53 activation in cancer cells (Figure 5D) and Isg15 deficient mice developed less lesions in the lung in a kRas mouse tumor model (Figure 5A&B). Furthermore, Isg15 depletion significantly enhances DNA damage-induced p53 activation in cancer cells (Figure 6). Thus, depletion of ISGylation may cooperate to induce p53 activity with the inhibition of ubiquitination induced by either DNA damage or Nutlin-3. Together, our study supports the hypothesis that ISGylation-mediated p53 degradation presents an alternative mechanism to regulate p53 stability and thus could be an attractive pathway for drug discovery.

## MATERIALS AND METHODS

### Animals, Cell culture conditions and treatments

All animal protocols used in this study were approved by the Institute of Molecular and Cell Biology Animal Safety and Use Committee. p53^−/−^ mice have been previously described[26]. Isg15^−/−^ mice were obtained from the Jackson Laboratory. Isg15^−/−^ p53^+/−^ were interbred to generate littermates with the following genotypes: Isg15^−/−^ p53^−/−^ and Isg15^−/−^. Mouse embryo fibroblasts (MEFs) were purified from 12.5d embryos.

HEK293T cells, HCT116 cells, and HCT116 Flag-tagged p53 cells were maintained in Dulbecco's modified Eagle's medium, and MCF7 cells in RPMI medium supplemented with 10%fetal bovine serum (Hyclone), 100 U/ml of penicillin, and 100 g/ml of streptomycin (Gibco). Cells were treated with Nutlin-3 (3 or 10 μM), or MG132 (25 μM), and then harvested when appropriate.

The inducible system for Src activity was described previously (21). The Src (8A7F)-mCherry-expressing construct, a gift from Dr. Frederic Bard (Institute of Molecular and Cell Biology, Singapore), was transduced with lentivirus in HCT116 cells expressing endogenous Flag-tagged p53. The cells expressing Src (8A7F) were selected based on mCherry expression. Imidazole (5 mM) was used to activate Src activity.

### Plasmids and siRNA transfection

Plasmids expressing hemagglutinin (HA)-tagged full-length p53 were gifts from Dr.Sheau-Yann Shieh (Institute of Biomedical Sciences,Taiwan). Plasmids expressing Myc-tagged Isg15, FLAG-tagged Herc5, HA-tagged Ube1L and UbcH8 were gifts from Dr.Keh-Chuang Chin (Genome Institute of Singapore, Singapore). Full-length Isg15 was cloned into the BamH1 and Xba1 sites of pcDNA3.1/His vector (Invitrogen) All point and deletion mutants were generated by PCR-based site-directed mutagenesis. Plasmids were introduced into cells using calcium phosphate transfections.

Transfection of siRNA was performed using calcium phosphate precipitation in HEK293T cells and Oligofectamine (Invitrogen) in other cell lines. The following SMART pool siRNAs were purchased from Dharmacon: p53 (5'-GAAAUUUGCGUGUGGAGUA-3', 5'-GUGCAGCUGUGGGUUGAUU-3', 5'-GCAGUCAGAUCCUAGCGUC-3', 5'-GGAGAAUAUUUCACCCUUC-3'), Isg15 (5'-GCAACGAAUUCCAGGUGUC-3', 5'-GCAGAUCACCCAGAAGAUU-3', 5'-GCACCGUGUUCAUGAAUCU-3', 5'-GGACAAAUGCGACGAACCU-3').

### Cell lysis, immunoprecipitation,and immunoblotting

Cells were lysed in TEGN buffer (10 mM Tris, pH 7.5, 1 mM EDTA, 420 mM NaCl, 10% glycerol, and 0.5% Nonidet P-40) containing 1 mM dithiothreitol (DTT) and protease inhibitors cocktail (Roche). For immunoprecipitation, the lysates were diluted with an equal volume of TEG buffer (10 mM Tris, pH 7.5,1 mM EDTA, and 20% glycerol) and incubated with antibodies and 15μl of 50% protein G beads (Pierce) for 1.5 h at 4°C. The immunoprecipitates were washed three times in 10S buffer (50 mM HEPES, pH 7.9, 250 mM NaCl, 0.2% NP-40, 0.1% Triton X-100, and 0.01% sodium dodecyl sulphate (SDS), then boiled in a protein sample buffer (2 M β-mercaptoethanol, 12% SDS, 0.5 M Tris, pH 6.8, 0.5 mg/ml bromophenol blue, and 30% glycerol), and analyzed by SDS-polyacrylamide gel electrophoresis (PAGE) followed by Western blotting. Antibodies used were the following: PAb1801 (sc98; Santa Cruz), p53 rabbit antibody (no. 9282; Cell Signaling ISG15 (AP1150a; ABGENT), phosphotyrosine (610012; BD Biosciences), p21 (OP79; Oncogene Science), actin (A2066; Sigma), HA (16B12; Covance), His (sc8036; SantaCruz), GST (RPN1236; Amersham), FLAG M2 antibody (Sigma), and FLAG rabbit antibody (no.2368, Cell Signaling).

### Analysis of the conformational form of p53 protein

p53 conformational form was analysed by immunoprecipitation (IP). Cells were lysed in TEGN buffer containing protease inhibitors cocktail (Roche). For IP, the lysates were diluted with an equal volume of TEG and incubated with conformation-specific antibodies, PAb1620 (Calbiochem), or PAb240 (Calbiochem), and 15μl of 50% protein G beads (Pierce) for 1.5 h at 4°C. The immunoprecipitates were washed three times in IP buffer (TEGN: TEG=1:1), and then boiled in a protein sample buffer, and analyzed by SDS-polyacrylamide gel electrophoresis (PAGE) followed by Western blotting. The p53 in the immunoprecipitates was detected by p53 Sheep PAb (Ab-7) (Calbiochem).

### *In vitro* kinase assay

For *in vitro* kinase assays, purified recombinant His-p53 fusion proteins were incubated with GST-Src fusion proteins in kinase buffer (20 mM Tris, pH 7.5, 10 mM MgCl2, 2 mM MnCl2, and 1 mM DTT) supplemented with 50 μM ATP at 30°C for 15 min. The reaction was stopped by the addition of 0.5 volume of protein sample buffer, and the proteins were resolved by SDS-PAGE. The phosphorylation of p53 and Src was visualized by Western blotting using anti-phospho-Tyrosine antibodies.

### Ni-beads pulldown assays

Transfected cells for different experiments were harvested and sonicated in buffer A (6 M guanidine-HCl, 0.1 MNa_2_HPO_4_/NaH_2_PO_4_, 10 mM imidazole, pH 8.0). Cell lysates were shaked with 30μl of 50% Ni-beads (Qiagen) at room temperature for 1 h. The precipitated beads were washed once in buffer A, once in buffer TI (20 mM imidazole, 0.2%Triton X-100, 25 mMTris-HCl, pH 6.8) mixed with buffer A (1:3), and three times in TI buffer. Proteins bound to beads were boiled in sample buffer and analysed by SDS-PAGE followed by Western blotting.

### EdU incorporation

DNA replication was measured using a Click-it EdU assay kit, which is based on incorporation of the thymidine analogue 5-ethynyl-2'-deoxyuridine (EdU) into DNA during replication (Invitrogen). Then, 10 μM EdU was added to the cell culture medium 30 min before the cells were harvested and fixed in 4% paraformaldehyde. EdU was detected by flow cytometry after it reacted with Alexa647-azide (Invitrogen).

### MTT assay and Colony forming Assay

For measuring cell proliferation, the MTT assay was carried out. 2X10^3^ cells transfected with siRNA per well were seeding on 96-well plates and cultured for different periods. At the end of the assay time, 10 μl of MTT solution (5 mg/ml, Invitrogen) was added to each well, and then incubated for 4 h at 37°C. After removing the cultured medium, 100 μl of DMSO was added to each well, and the plates were read at 540 nm using a spectrophotometric plate reader with a reference wavelength at 650 nm.

For analysis of the role of ISG15 in colony forming assay, cells were transfected with control of ISG15 siRNAs using Oligofectamine (Invitrogen). For DNA damage response, 3000 cells/well were seeding onto 35 mm dish after treatment with 1Gy of X-ray. Two weeks later colonies were fixed in 70% ethanol and stained by 1% crystal violet solution before counting.

### Cell transformation, soft agar assay and tumorigenesis assay

Mouse embryonic fibroblasts (MEFs) were isolated from embryonic 12.5 day-old mouse embryos. MEFs were transfected using V-Src viral particles and selected for resistance to neomycin for 2-3 week.

For soft agar assay, the V-Src-transformed MEFs (5 × 10^3^) were resuspended in 0.3% low melting agarose (Invitrogen) in DMEM supplemented with 10% FBS and then overlaid on 0.6% low melting agarose in the same medium in 35mm dishes. The dishes were incubated at 37°C for 3 weeks. At the end of the assay time, 1 ml of MTT solution (1 mg/ml, Invitrogen) was added to each well, and then incubated for 4 h at 37°C, and then colonies that stained by MTT were counted.

For *in vivo* tumorigenesis assay, the V-Src-transformed MEFs (1 × 10^4^) that mixed with Matrigel (BD Biosciences) were subcutaneously injected into nude mice. 21 days after injection, mice were sacrificed, and tumors were collected and weighed.

### RT-PCR

Total RNA from the cells was extracted by TRIzol (Invitrogen), and the synthesis of cDNA was performed using an oligo(dT) primer by ReverAid^™^ Premium First Strand cDNA Synthesis Kit (Fermentas Life Science), according to the manufacturers' protocols. The quantification of gene transcripts was analysed by real-time PCR using SYBR green dye (KAPABIOSYSTEMS). All values were normalized to the expressing level of Actin. The primers were as follows:

For human samples: Actin forward, CCAGAGGAAGAGAGGCATCC; Actin reverse, GTGGTGGTGAAGCTGTAGCC. V-Src forward, TTCGGGGACTTCAACACTTC; V-Src reverse, ATGAGCCAGCCACCAGTTAC; H-Ras forward, TGCCATCAACAACACCAAGT; H-Ras reverse, ATCTCACGCACCAACGTGTA.

For mouse sample, p21 forward, AAGTGTGCCGTTGTCCTTC; p21 reverse, ACTTCAGGGTTTTCTCTTGC Actin forward, AGCCATGTACGTAGCCATCC; Actin reverse, CTCTCAGCTGTGGTGGTGAA.

### Cell fractionation

The cell pellets were resuspended in hypotonic buffer (20 mM Tris, 10 mM NaCl, 3 mM MgCl_2_, 10% glycerol) with proteases inhibitor cocktail (Roche), phosphatase inhibitor (Roche), and incubated on ice for 15 min. 25% NP-40 was then added to a final concentration of 0.5% and the cells were vortexed for 10 seconds at highest setting. The cell lysates were centrifuged at 5000 rpm for 5 min to obtain nuclear pellets. Supernatants were collected as cytosolic fractions. Nuclear pellets were washed with hypotonic buffer, and resuspended in TEGN buffer with protease inhibitor and phosphatase inhibitor, and incubated on ice for 15 min. Nuclear extracts were cleared by centrifugation, and the supernatants were collected as nuclear fraction.

### Statistical analysis

Values are mean +/− SEM. Comparison of mean values between group was evaluated by 2-tailed Student't t-test or ANOVA where indicated. A p-value less than 0.05 was considered significant.

## SUPPLEMENTARY MATERIAL AND FIGURES


