# Supplementary material for: Oncogene-mediated regulation of p53 ISGylation and functions
Source: Oncotarget. 2014 Jul 11;5(14):5808–18. doi: 10.18632/oncotarget.2199 (PMC4170631; doi:10.18632/oncotarget.2199)
Supplement: Supplementary file 1 [file oncotarget-05-5808-s001.pdf]

Oncogene-mediated regulation of p53 ISGylation and functions

Supplementary Material

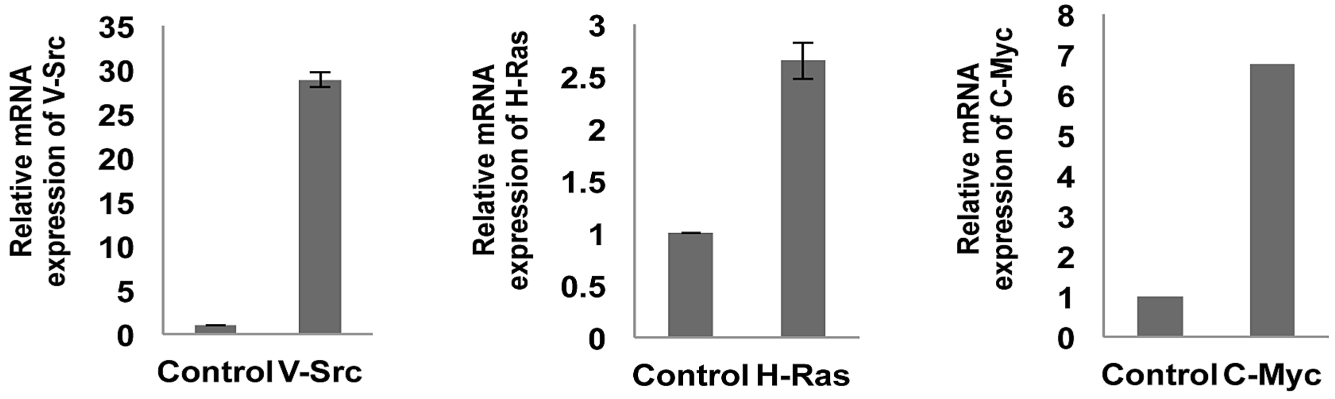

Figure S1: The graphs are shown that the expression level of transfected V-Src, H-Ras, and C-Myc in 293T cells.

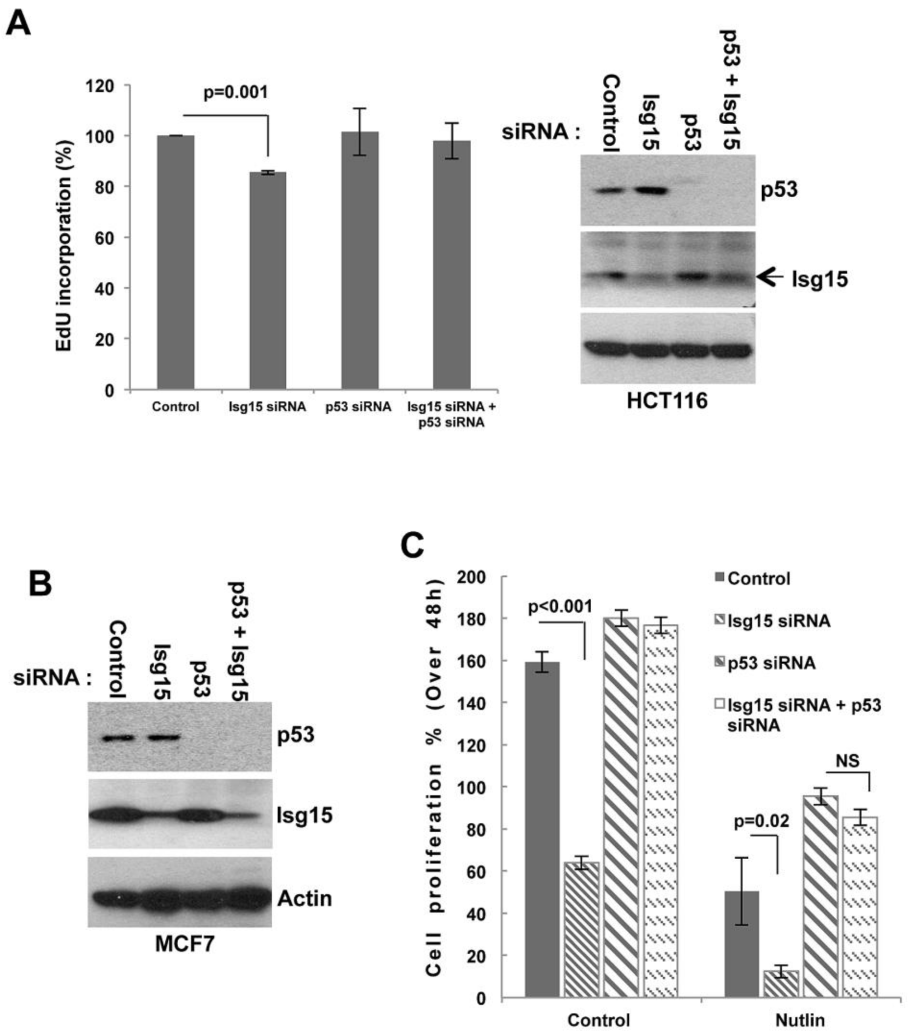

Figure S2: Knockdown of Isg15 enhances p53 activity in HCT116 cells. (A) Knockdown of Isg15 decreases DNA replication. HCT116 were transfected with either control, Isg15 siRNA, p53 siRNA or a combination of siRNAs. The cells were labelled with 10 μM EdU for 30 min before analysis. Knockdown efficiency of Isg15 and p53 were shown in right panel. (B) Knockdown efficiency of Isg15 and p53 in MCF7 cells were analyzed by Western blotting. (C) Knockdown of Isg15 enhances the Nutlin-mediated inhibition of cell proliferation. MTT assay were performed with HCT116 cells transfected with either control, Isg15 siRNA,

p53 siRNA or a combination of siRNAs in the absence or presence of Nutlin (3 μM). The difference of proliferating activity between 24h to 72h after seeding was shown in graphs.

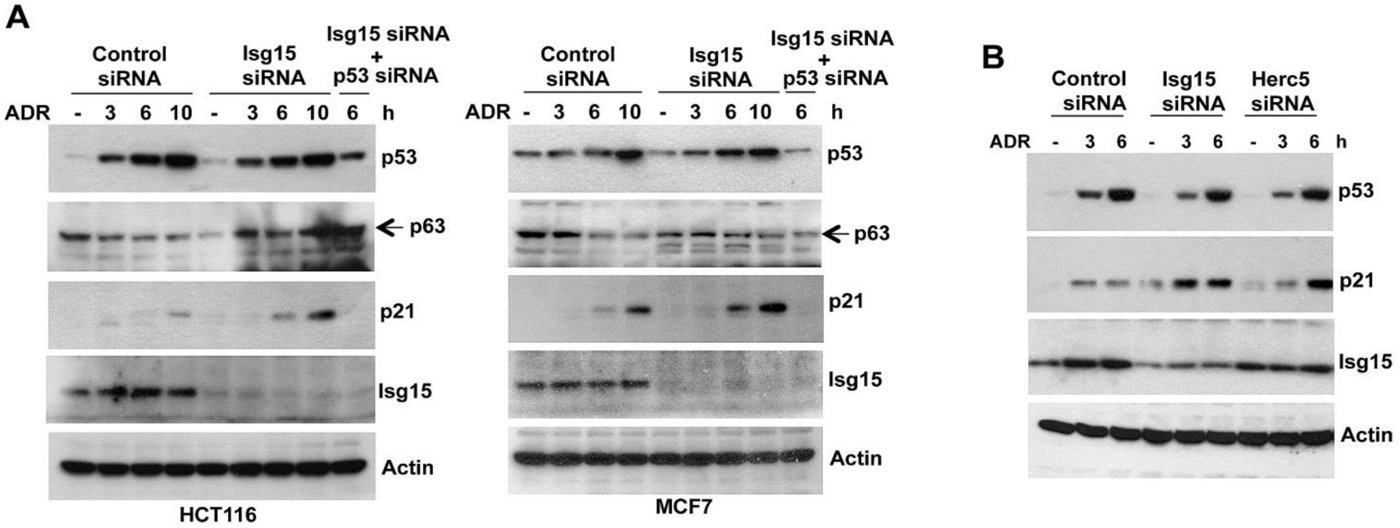

Figure S3: Isg15 regulates p53-dependent DNA damage response. (A) Isg15 regulates Adriamycin-induced p53 response. HCT116 cells and MCF7 cells were transfected with control or Isg15 siRNAs, or together with p53 siRNA. . Cells were treated with Adriamycin and collected at the time points indicated. (B) Knockdown of Herc5 has the similar effect to Isg15 depletion. HCT116 cells were transfected with control, or Isg15 siRNAs, or Herc5 siRNA. Cells were treated with Adriamycin and collected at the time points indicated. Cell lysates were analysed by Western blotting using indicated antibodies.
